# Beta-lactam antibiotic test doses in the emergency department

**DOI:** 10.1016/j.waojou.2019.100093

**Published:** 2020-01-07

**Authors:** Michelle Maguire, Bryan D. Hayes, Lanting Fuh, Ramy Elshaboury, Ronak G. Gandhi, Sarah Bor, Erica S. Shenoy, Anna R. Wolfson, Christian M. Mancini, Kimberly G. Blumenthal

**Affiliations:** a Department of Pharmacy, Massachusetts General Hospital, Boston, MA, USA; b Harvard Medical School, Boston, MA, USA; c Division of Infectious Diseases, Department of Medicine, Massachusetts General Hospital, Boston, MA, USA; d Infection Control Unit, Massachusetts General Hospital, Boston, MA, USA; e Division of Rheumatology, Allergy, and Immunology, Department of Medicine, Massachusetts General Hospital, Boston, MA, USA; f Mongan Institute, Massachusetts General Hospital, Boston, MA, USA

**Keywords:** Challenge, Penicillin, Hypersensitivity, Acute, ED, emergency department, HSR, hypersensitivity reaction, PST, penicillin skin testing, EHR, electronic health record, CI, confidence interval

## Abstract

**Background:**

Facilitating beta-lactam antibiotic use in patients reporting beta-lactam allergies in acute care settings is important to individual patient outcomes and public health; however, few initiatives have targeted the Emergency Department (ED) setting.

**Methods:**

We implemented pathways for patients reporting prior penicillin and/or cephalosporin hypersensitivity as part of a hospital guideline in the ED of a large academic medical center in the United States. We described beta-lactam test doses, pathway compliance, hypersensitivity reactions (HSRs), and allergy record updating associated with ED-administered beta-lactam test doses from October 2016 to June 2018.

**Results:**

310 beta-lactam antibiotic test doses were administered to patients with penicillin and/or cephalosporin allergy histories in the study period (average volume 15/month [standard deviation 4]). Test doses were to cephalosporins (85%), penicillins (12%), and carbapenems (4%). 219 (71%) of test doses were compliant with the pathways. Ten patients (3.2%; 95% CI 1.6%–5.9%) had HSRs; five HSR patients (50%) had beta-lactams administered that were not pathway compliant. The allergy record was updated in 146 (47%) of patients, with improvement over the study period (p < 0.001).

**Conclusions:**

Inpatient approaches to prescribing beta-lactams in patients reporting beta-lactam allergies can be operationalized in the ED. Additional efforts are required to ensure guideline compliance and appropriate allergy documentation.

## Introduction

Beta-lactam antibiotic allergies are commonly reported, but uncommonly reflective of clinically significant immunologic reactions.^1^ Unconfirmed beta-lactam allergies can compromise quality of care; alternative antimicrobials may be less effective and carry a higher risk of adverse sequelae (e.g., *Clostridioides difficile* infections, adverse drug reactions, death).^1^, ^2^, ^3^

Although penicillin skin testing (PST) can be used in patients reporting beta-lactam allergies to optimize antibiotic use in acute care settings,^4^ including the emergency department (ED),^5^ PST can be time-consuming and is not necessary prior to administering most cephalosporins,^6^ antibiotics commonly used in the emergency and inpatient settings.^4^ Direct amoxicillin challenge protocols have been able to rule out penicillin allergy in patients at low-risk for having true penicillin allergy in the ED,^7^ but this process consumes a few hours of ED time that may result in unacceptable delays in therapeutic antibiotic treatment.

**Fig. 1:**
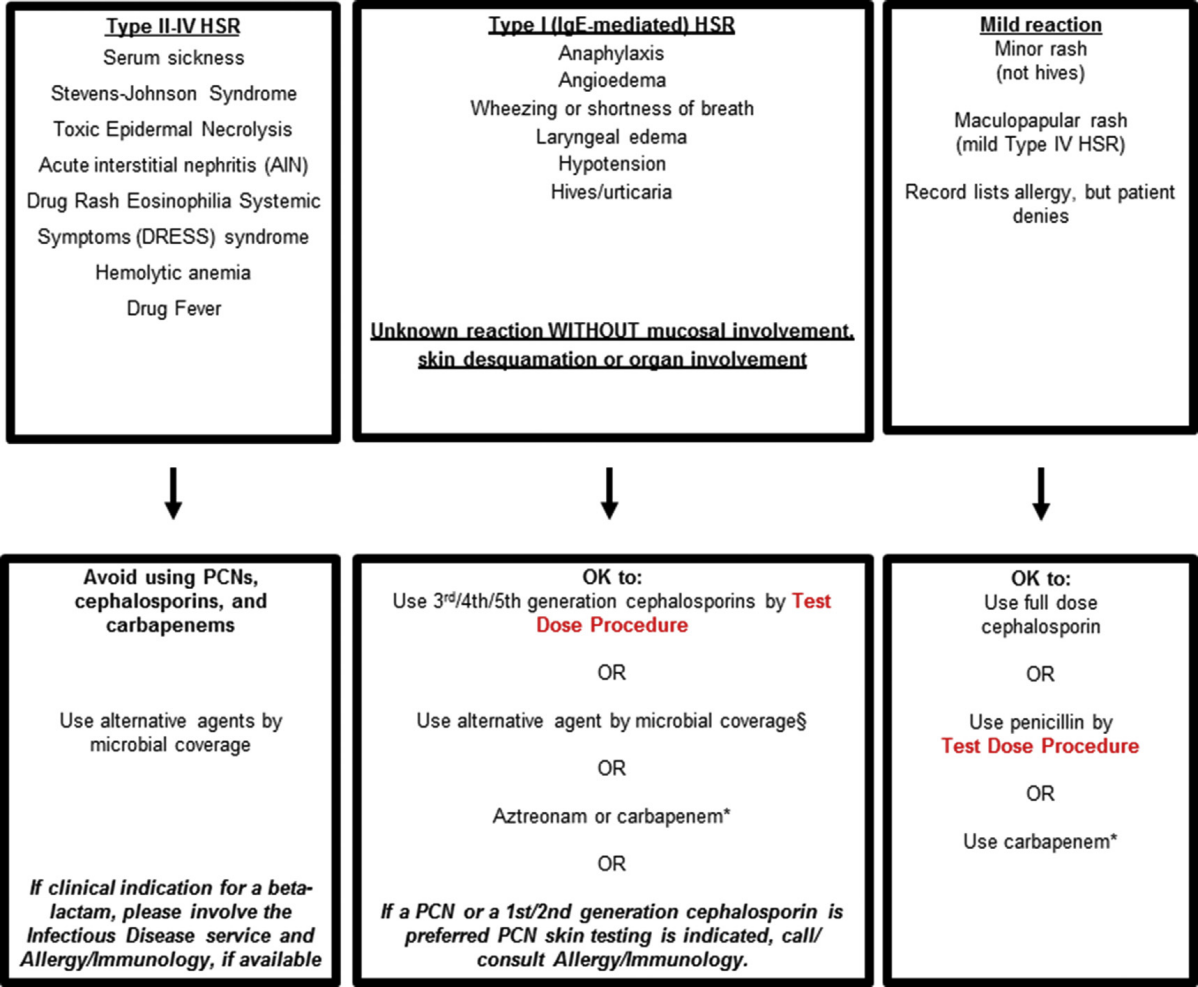

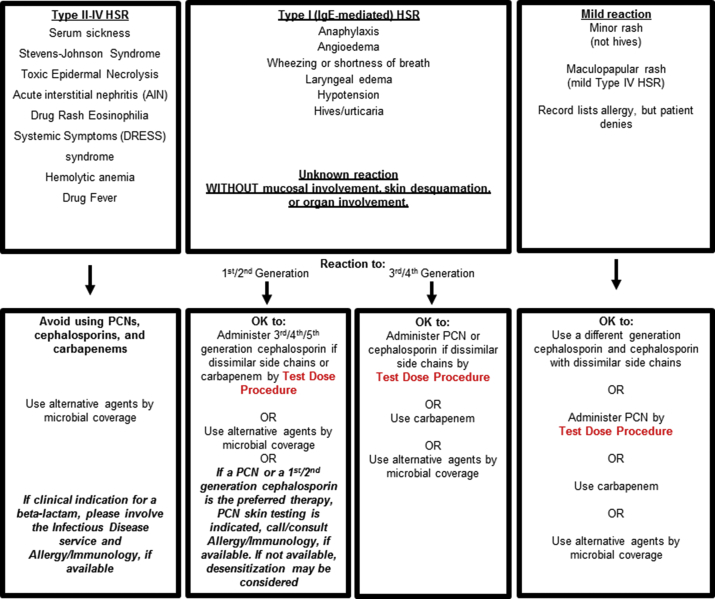
Penicillin and Cephalosporin Hypersensitivity Pathways. The pathways consider actions based on the patient'd allergy history considering three groups: (1) Type II-IV hypersensitivity reaction (HSR), (2) Type I, IgE-mediated HSR or unknown reaction, and (3) Mild reactions. The test dose procedures are uniformly 2-steps where 10% of the beta-lactam antibiotic is initially given; if there is no reaction after 1-hour of observation, then 100% of the beta-lactam antibiotic (i.e., one full therapeutic dose) is given with another hour of observation. All hemodynamically stable patients with a history of penicillin and/or cephalosporin allergy requiring a beta-lactam antibiotic were eligible for test dose procedures. If possible, beta-blockers were held the day of test dose procedures. PST is required for patients with a Type I (IgE-mediated) HSR to a penicillin or 1st/2nd generation cephalosporin or unknown beta-lactam allergy history who are being considered for treatment with a penicillin or 1st/2nd generation cephalosporin only (middle box). A cephalosporin hypersensitivity pathway was also implemented for patients with cephalosporin allergy histories. These inpatient pathways were piloted in the Emergency Department of one acute care hospital in 2016 after education by clinical pharamacists.

Penicillin and cephalosporin hypersensitivity pathways were designed to safely increase beta-lactam antibiotic use in hospitalized patients with histories of penicillin and/or cephalosporin allergy (Fig. 1).^8^ These pathways were studied at two academic medical centers in Boston,^8^^,^^9^ and spread across 5 acute care hospitals that are part of a large healthcare system in the greater Boston, Massachusetts, USA area.^10^^,^^11^ The pathways were implemented in all inpatient areas, including pediatrics and obstetrics, and served as antibiotic prescribing guidelines for non-allergist providers. The pathways use standardized 2-step test doses to the desired therapeutic beta-lactam antibiotic, often without preceding PST (Fig. 1). The pathways were associated with an approximately 2-fold increased beta-lactam use and were safe; few reactions required intramuscular epinephrine.^9^^,^^11^ In this study, we assessed the penicillin and cephalosporin hypersensitivity pathways in the ED setting, describing pilot pathway implementation of test doses, including pathway compliance, hypersensitivity reactions (HSRs), and allergy documentation.

## Methods

The inpatient penicillin and cephalosporin hypersensitivity pathways were implemented in the ED of one acute care hospital in 2016 with pharmacist-led education. Test doses were ordered by an ED healthcare provider, including medical doctors, nurse practitioners, and physician assistants. After pharmacist verification and preparation of the test dose procedure order, ED nurses administered test doses and performed the requisite observation.

We identified all patients who had a history of penicillin and/or cephalosporin allergy and received a beta-lactam antibiotic test dose in the ED between October 2016 and June 2018. Electronic health record (EHR)-collected data included patient demographics, allergy history, and test dose outcomes. The test dose outcomes included HSR signs, symptoms, and treatment and allergy documentation updates. HSRs from the beta-lactam antibiotics included reactions that were potentially immunologic such as urticaria, angioedema, anaphylaxis, shortness of breath, and reactions concerning for organ specific reactions or severe cutaneous adverse reactions. Compliance with pathways was assessed retrospectively by a clinical pharmacist and allergist/immunologist. If there was not adequate documentation to support compliance with the pathways, noncompliance was assumed. We used a chi-squared test to compare frequencies of appropriate allergy documentation.

## Results

We identified 310 patients (mean age 63 years [standard deviation (SD) 19 years] and 38% male) who received a beta-lactam antibiotic by test dose procedure in the ED. Test doses occurred on average 15 times per month (SD 4). Test doses were to cephalosporins (n = 262, 85%), penicillins (n = 37, 12%), and carbapenems (n = 11, 4%).

The pathways were followed in 219 (71%) cases overall. Among noncompliant cases (n = 91, 29%), most (n = 60, 66%) unnecessarily administered a test dose when a full-dose was appropriate (e.g., mild reaction, Fig. 1 rightmost box). A common pathway deviation was administration of a first or second generation cephalosporin test dose in patients with a type I or unknown beta-lactam allergy history without first consulting an allergist/immunologist for PST (n = 14, 16%). In a few cases (n = 3, 3%), a test dose was administered when there was a suspected type II-IV HSR. Of noncompliant cases, 85 (93%) were performed at the discretion of the emergency medicine clinician and 6 (7%) were guided by specialist consulting services.

In all, 10 patients (3.2%; 95% CI 1.6%–5.9%) had HSRs (Table 1); 5 (50%) reacted at the test dose step, and 5 (50%) reacted at the full dose step or with subsequent therapeutic doses. Five HSR patients (50%) had beta-lactams administered that were not compliant with the pathways; all were performed at the discretion of the emergency medicine clinician.

**Table 1:** Test doses resulting in hypersensitivity reactions

Compliant with Pathway	Beta-Lactam Allergy	Beta-Lactam Historical Reaction	Test Dosed Beta-Lactam	Test Dose Reaction	HSR Treatment^a^
Yes	PCN	Hives/SOB	Ceftriaxone	SOB/Throat Swelling	Antihistamine
	PCN	Rash	Ampicillin/Sulbactam	Hives	Antihistamine
	Amoxicillin	Throat Closing/Dyspnea	Ceftriaxone	Throat Closing Sensation	Antihistamine
	Ceftriaxone	Rash	Piperacillin/Tazobactam	Hypotension/Swelling	Antihistamine
	CPH	Rash	Ceftazidime	AIN	Antihistamine
No	PCN	Hives	Ampicillin/Sulbactam	Rash	Antihistamine
	PCN	Anaphylaxis	Cefoxitin	Anaphylaxis	Antihistamine, Corticosteroid, and Epinephrine
	PCN	Rash	Meropenem^b^	Rash	Antihistamine
	PCN	Unknown	Ampicillin/Sulbactam	Undocumented	Antihistamine
	Ampicillin	Rash	Cefepime^b^	Rash	None

*Abbreviations*: PCN, penicillin; SOB, shortness of breath; CPH, cephalosporin; AIN, acute interstitial nephritis.

a All HSR patients had the culprit beta-lactam discontinued in addition to the specified anti-allergic treatments

b Patient test dose was not necessary according to the penicillin hypersensitivity pathway (Fig. 1).

The allergy list was appropriately updated in 146 (47%) of patients overall, with improvement observed over the study period (from 18% in month one to 71% in month 21 p < 0.001). Allergy updating was more frequent for the patients with HSRs; 9 (90%) of HSR patients had their beta-lactam allergy appropriately added.

## Discussion

We reviewed 310 beta-lactam antibiotic test doses performed in the ED for patients with reported allergies to penicillins or cephalosporins. Most test doses were for cephalosporins, and while HSRs were uncommon, half of the HSRs were triggered by the test dose. The pathways were followed in at least 71% of cases. Although overall allergy documentation was poor, it improved with time, potentially as the pathways became more familiar.

Patients with penicillin allergies can and should be evaluated in all healthcare settings, but the ED presents unique operational challenges given that interventions must occur 24 hours a day, including weekends and holidays, when specialist access may be restricted. Further, patients in the ED have varied levels of acuity and lengths of stay; while some patients are ultimately admitted and need parenteral antibiotics, others are discharged home on oral antibiotics. For acutely infected patients, delays in antibiotic treatment can result in deleterious outcomes; one study documented that patients with a penicillin allergy history had a longer mean time to first antibiotic dose than those without a penicillin allergy history (236.1 minutes vs 186.6 minutes, p = 0.03).^12^ As such, interventions aimed at clarifying the underlying penicillin allergy, with PST and/or amoxicillin challenge, should not delay optimal therapeutic treatment. The resources available in the ED setting, including nurse staffing and easily accessed anti-allergic medications, make it an ideal location for test dose challenges to the therapeutic antibiotic; it was not surprising that the ED embraced this approach and performed an average of 15 beta-lactam test doses per month in this 21-month period.

The observed HSR frequency from the penicillin and cephalosporin pathways in the ED setting (3.2%) was similar to HSR frequency observed in our largest system analysis to date (3.8%).^11^ In this study, more patients reacted at the test dose step (50%), compared to our prior study when just 33% of HSRs occurred at the test dose step.^11^

Although the majority of ED test doses followed the established hypersensitivity pathways, more than one-quarter were noncompliant, and half of the HSRs occurred in patients receiving a noncompliant test dose. Adherence in this study was notably lower than observed in the initial inpatient analyses of these pathways where 91% of test doses were guideline-compliant and no adverse reactions occurred from non-compliant test doses.^8^ While occasionally pathway deviations were directed by specialists, including allergy specialists, most deviations were at the direction of the emergency medicine clinician. ED providers may need increased education and/or routine compliance checking by the clinical pharmacist.

Updating of a patient's allergy record is important to the quality and safety of patient care.^13^ Accurate allergy documentation after beta-lactam test doses allows for clinicians to quickly identify antibiotics tolerated in the past and potentially avoid the administration of an unnecessary test dose procedure on a future medical encounter. While overall EHR allergies were updated in less than half of patients who received a beta-lactam antibiotic test dose, the frequency of allergy updates in the EHR more than tripled in the second half of study period, an improvement internally attributed to increased comfort with the pathways and educational initiatives between ED clinicians and pharmacists. Targeted educational efforts continue to be particularly important because patients may not be cared for in the ED long enough to benefit from the embedded clinical decision support designed to improve documentation after beta-lactam antibiotic test doses.^14^

### Limitations

We reviewed patients who received a beta-lactam test dose in the ED setting, but were unable to identify how many ED patients in this time-frame were eligible to receive beta-lactam test doses. Thus, we cannot completely judge the success of our implementation. Because this study was retrospective, we relied on EHR documentation that is prone to errors and omissions. To decrease miscategorization, many parts of the EHR were evaluated, including clinician and nurse documentation and medication administration records. While we were limited by sample size from doing comparative analyses, we anticipate that these initial data may motivate expansion of these pathways more broadly into ED settings across our healthcare system, and potentially to other institutions that have adopted similar approaches.

## Conclusion

We describe our experience using a guideline with penicillin and cephalosporin hypersensitivity pathways in the ED setting. ED test doses through the guideline were feasible and HSRs infrequent, but we found lower guideline compliance and recognized persistent allergy documentation challenges that require unique solutions given that the ED has quick patient throughput and a large and diverse provider pool. Still, pathway implementation facilitated standardized beta-lactam antibiotic use in patients with beta-lactam allergy histories in a timely and safe manner, likely improving the quality and safety of patient care.

## Funding

This work was supported by Partners Quality, Safety, and Value and the Partners Clinical Process Improvement Leadership Program (CPIP). Dr. Blumenthal received career development support from the NIH K01AI125631, the American Academy of Allergy Asthma and Immunology (AAAAI) Foundation, and the MGH Claflin Distinguished Scholar Award. The content is solely the responsibility of the authors and does not necessarily represent the official views of the National Institutes of Health, AAAI Foundation, nor MGH.

## Conflicts of interest

KGB and ESS report a licensed clinical decision support tool for inpatient beta-lactam allergy evaluation. The remaining authors have nothing to disclose.

## Consent for publications

All authors confirm their consent for publication.

## Ethics approval

This study protocol was approved by the Partners Human Research Committee protocol 2015P001873.
